# Paleontological Puzzles, Neurological Solutions: How Dinosaur Collagen Analysis Advanced X-ray Scanning for TBI

**DOI:** 10.1063/4.0000973

**Published:** 2025-10-27

**Authors:** Joseph Orgel, Rama Sashank Madhurapantula

**Affiliations:** Illinois Tech, Chicago, Il, USA

## Abstract

We analyzed the structural context of peptides recovered from dinosaur fossils, including *Tyrannosaurus rex*, using X-ray diffraction data obtained directly from intact fossils and dissected fossil tissues. By comparing this fossil data with diffraction patterns from extant collagen and mapping the ancient peptides onto molecular models derived from modern collagen microfibrils, we gained insights into preservation patterns within the collagen D-period. This multi-faceted diffraction approach contextualized the survival of collagenous components by correlating preserved peptide locations with the detailed 3D fibril architecture. Furthermore, the data confirmed preserved nanoscopic tissue structure within the fossils, allowing comparison with contemporary collagen arrangements. Critically, the challenges inherent in analyzing these unique fossil samples spurred advancements in X-ray scanning methodologies. These enhanced techniques subsequently enabled novel approaches to understanding white and gray matter damage signatures in traumatic brain injury, demonstrating an unexpected link between paleontological research and modern neuroscience.

